# Sequencing-based genome-wide association studies reporting standards

**DOI:** 10.1016/j.xgen.2021.100005

**Published:** 2021-10-13

**Authors:** Aoife McMahon, Elizabeth Lewis, Annalisa Buniello, Maria Cerezo, Peggy Hall, Elliot Sollis, Helen Parkinson, Lucia A. Hindorff, Laura W. Harris, Jacqueline A.L. MacArthur

**Affiliations:** 1 European Molecular Biology Laboratory, European Bioinformatics Institute, Wellcome Genome Campus, Hinxton, UK; 2 Division of Genomic Medicine, National Human Genome Research Institute, National Institutes of Health, Bethesda, MD 20892, USA; 3 BHF Data Science Centre, Health Data Research UK, London, UK; 4 Lead contact

## Abstract

Genome sequencing has recently become a viable genotyping technology for use in genome-wide association studies (GWASs), offering the potential to analyze a broader range of genome-wide variation, including rare variants. To survey current standards, we assessed the content and quality of reporting of statistical methods, analyses, results, and datasets in 167 exome- or genome-wide-sequencing-based GWAS publications published from 2014 to 2020; 81% of publications included tests of aggregate association across multiple variants, with multiple test models frequently used. We observed a lack of standardized terms and incomplete reporting of datasets, particularly for variants analyzed in aggregate tests. We also find a lower frequency of sharing of summary statistics compared with array-based GWASs. Reporting standards and increased data sharing are required to ensure sequencing-based association study data are findable, interoperable, accessible, and reusable (FAIR). To support that, we recommend adopting the standard terminology of sequencing-based GWAS (seqGWAS). Further, we recommend that single-variant analyses be reported following the same standards and conventions as standard array-based GWASs and be shared in the GWAS Catalog. We also provide initial recommended standards for aggregate analyses metadata and summary statistics.

## In brief

McMahon et al. report an analysis of the sequencing-based GWAS literature, finding a lack of standardized language and incomplete reporting, along with less-frequent sharing of summary statistics compared with that of array-based GWASs. We provide recommendations for the reporting and sharing of sequencing-based GWASs to increase FAIRness of these valuable datasets.

## Graphical Abstract

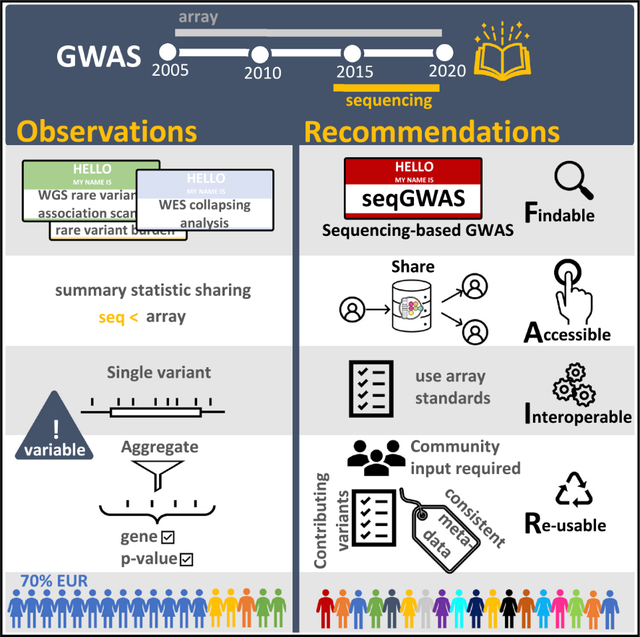

## INTRODUCTION

Huge advances in the field of human genetics can be attributed to the advent of genome-wide association studies (GWASs) more than 15 years ago.^1,2^ In recent years, decreasing costs and advances in analytic methods have made high-throughput whole-genome sequencing (WGS) and whole-exome sequencing (WES) feasible alternatives to array-based genotyping in GWASs.^3,4^ Sequencing offers a significant advantage over array-based methods, with the potential to detect and genotype all variants present in a sample, not only those present on an array or imputation reference panel. Most arrays are designed to assay common variants (minor allele frequency [MAF] > 5%), omitting rare (MAF < 1%) and low-frequency (MAF 1%–5%) variants. The analysis of these rarer variants could explain additional disease risk or trait variability and help overcome the problem of “missing heritability.”^5,6^ In addition, most arrays have historically been biased toward coverage of variation in European populations.^7^ The fact that sequencing potentially provides an unbiased assessment of variants within the population studied is particularly important for studies of non-European populations.^8,9^

There are challenges with analyzing many more and rarer variants. Single-variant tests, used as the standard in array-based GWASs, are typically underpowered when applied to low-frequency or rare variants, unless sample sizes or effects are very large. There are also issues with correcting for multiple testing when the number of statistical tests is very large. To address those issues, statistical methods have been designed specifically for rare-variant-association testing, which evaluate aggregate association over multiple variants in a genomic region (referred to here as “aggregate tests”).^10^ Variants are typically aggregated across biologically functional regions (e.g., a gene) with variants enriched for those likely to have larger effect sizes based on annotated or predicted functional effect (e.g., located in a splice junction or a predicted loss of function). The power of a particular aggregate test to detect an association will depend on how closely the model’s assumptions and contributing variants represent the true disease mechanism at each locus.

Repositories of scientific data have been indispensable in supporting research and in facilitating discoverability and integration across datasets through standard formats. The National Human Genome Research Institute-European Bioinformatics Institute (NHGRI-EBI) GWAS Catalog^11^ is the preeminent data resource of large-scale genetic-association studies, enabling research to identify causal variants, to understand disease mechanisms, and to establish targets for novel therapies.^12^ The GWAS Catalog infrastructure, data content, and standard formats have been designed to support array-based GWASs. Attempts to expand the scope of the Catalog to include sequencing-based association studies have been hindered by the need to develop new standards for the differences in methods, the metadata required to represent them, and the format of the results, particularly for aggregate analyses.

Here, we analyze the current landscape of published sequencing-based association studies to determine requirements for hosting and sharing those datasets in the GWAS Catalog and recommend best practices for reporting. First, we comprehensively reviewed publications reporting sequencing-based association studies, assessing the range of experimental designs and statistical methods, as well as the content and quality of reporting for analyses, methods, and datasets included in publications. We hope that this review will form a rallying point for building community consensus on standards. This work has also informed the development of the GWAS Catalog infrastructure and data-representation schema to support inclusion of sequencing-based association studies, which are now accepted for submission at the GWAS Catalog. Our work at the GWAS Catalog is focused on enabling broad data sharing and defining standards to ensure sequencing-based association study data are findable, interoperable, accessible, and reusable (FAIR).^13^

## RESULTS

### Finding sequencing-based association studies

In our review of research publications (STAR Methods), we observed that a wide range of terms are used to describe sequencing-based genome or exome-wide association studies. The term “GWAS” is rarely used, and we have not seen an equivalent standard term emerge (Figure S1). Combinations of terminology were used, related to (1) analysis of associations (e.g., rare variant association analysis, rare variant aggregate association analysis, association test, and genome-wide significant associations), (2) the allele frequency of the variants analyzed (e.g., common variant and rare variant), (3) the analysis type, either single variant (e.g., single variant and variant level) or aggregate with multiple variants (e.g., gene-based, region-based, aggregate, gene burden, collapsing analysis, gene-level association, gene-level signal, and collapsed-variant tests).

**Figure 1.**
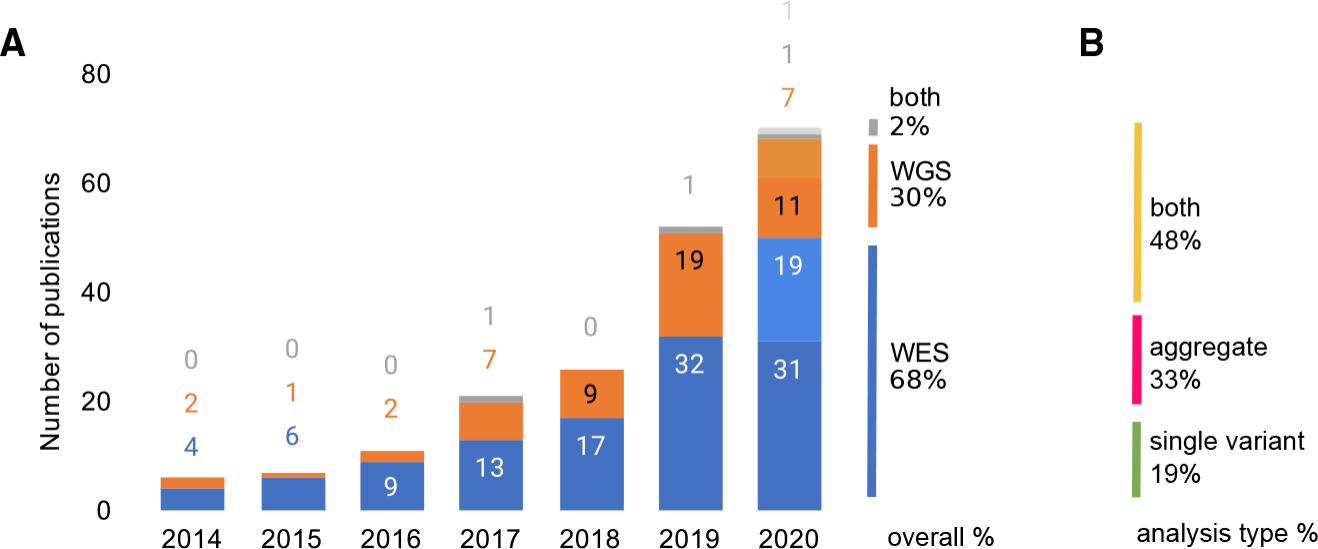
Sequencing-based GWAS publications, numbers, sequencing coverage, and analysis types (A) Number of sequencing-based association publications identified per year from 2014 to September both 2020, n = 167. Only genome-wide (and not limited to specific regions or subsets of genes) and population-based studies are included (see STAR Methods for more information). The final quarter of 2020 is projected based on the rate of growth in the final quarter of 2019 (projected data are presented in the light shade of each color) (B) The analysis types included in those publications. “Aggregate” refers to multi-variant analyses.

We identified 167 publications reporting genome-wide sequencing-based association analyses meeting our selection criteria (STAR Methods; Tables S1 and S2). The first study was published in 2014, with the number of publications increasing year after year to 2020 (Figure 1A). Because no standard terminology has been adopted for these studies, we were not able to search discriminately for sequencing-based association studies meeting our criteria, and permissive searches (e.g., for “WGS OR WES association”) yield too many results to feasibly review manually (Figure S2); therefore, we expect this to be an underestimate of publications reporting sequencing-based GWASs (seqGWAS). Most publications analyzed WES data only (68%), approximately one-third analyzed WGS data (30%), and some publications included both coverage types (2%) (Figure 1A). Many publications that used WES and WGS sequencing data limited their analyses to pre-specified regions of interest; those targeted analyses are not the focus of this work and were, therefore, excluded from the analysis.

### Association tests and qualifying variants

**Figure 2.**
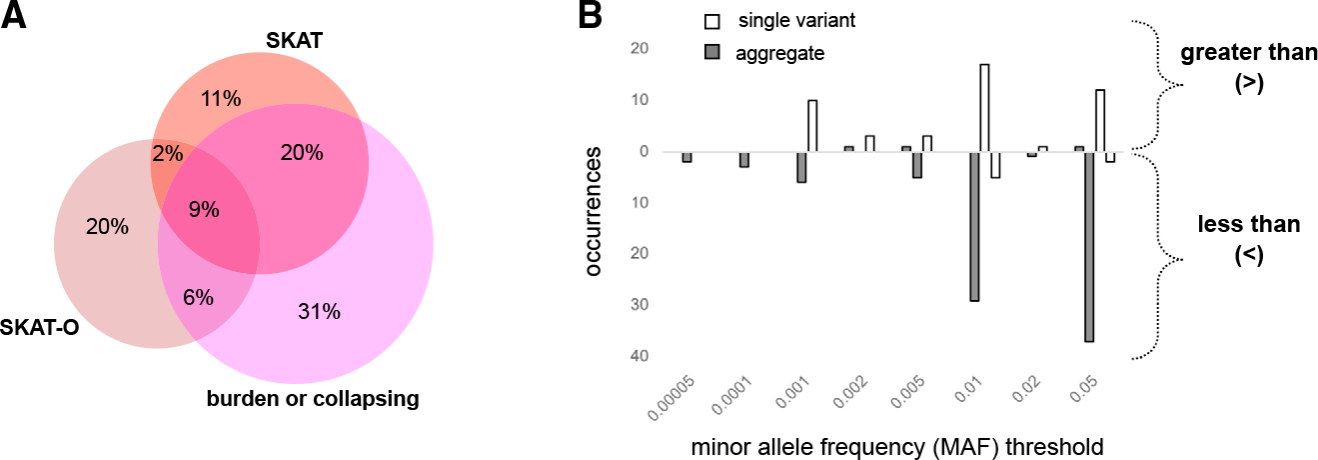
Statistical analysis methods used in sequencing-based GWAS publications (A) Overlap among methods used in aggregate-analysis publications. Of 65 publications that use either SKAT, SKAT-O, or a burden test, 40% use at least two methods. Text related to study design was extracted by experienced curators and searched for the terms “SKAT,” “SKAT-O,” and “burden” or “collaps*” (where * refers to a wildcard for searching). (B) Minor allele frequency thresholds used in single-variant and aggregate analyses. “Greater than or equal to” thresholds are displayed above the x axis; “less than or equal to” thresholds are displayed below the x axis. Thresholds were extracted from publications in which one or two thresholds were provided (single variant: n = 53 thresholds from 51 publications; aggregate: n = 86 thresholds from 77 publications). See Figure S4 for additional details on MAF-threshold reporting.

We surveyed the types of association tests included in these publications. Most frequent was the inclusion of both single-variant and aggregate analyses (48%), followed by aggregate analysis only (33%), and a minority of publications (19%) included single-variant analyses only (Figure 1B). Of the publications including aggregate tests, a wide range of statistical models and tools were used, with publications commonly using multiple models. For example, of publications that used one of the three most-common aggregation methods^10^ (burden/collapsing, variance-component [SKAT], and combined burden and variance-component [SKAT-O] tests), 40% (n = 65) used at least two of those methods (Figure 2A). The language used to describe those methods is varied; for example, SKAT is referred to variously as kernel based, dispersion based, or variance-component based (Figure S3).

We also examined variant-filtering or “masking” approaches. Minor allele frequency thresholds were reported in 72% of single-variant and 84% of aggregate-analysis publications, with the remainder either not reporting any MAF threshold or using all variants (26% of single variant/16% of aggregate) (Figure S4). “Greater than” thresholds were typically used for single-variant analysis, with 57% of analyses employing a MAF threshold of 0.01 or greater, limiting those analyses to the common variant space (Figure 2B) (n = 30/53 thresholded analyses from 51 publications). In contrast, aggregate analyses typically employed “less than” thresholds, to include only low-frequency (<0.05), rare (<0.005), or ultra-rare variants. Most aggregate analyses used <0.01 or <0.05 thresholds (78%, n = 67/86 thresholded analyses from 77 publications).

Many publications (63%, n = 75/120) also performed analyses on variants with predicted biological effect. Authors filtered for predicted functional effect based on transcript annotation (e.g., using the Variant Effect Predictor^14^) or protein structure (e.g., using Sorting Intolerant from Tolerant [SIFT],^15^ Polymorphism Phenotyping v2 [PolyPhen]^16^ and combined annotation-dependent depletion [CADD]^17^) or based on measures of evolutionary conservation or variation intolerance.^18,19^ An analysis of the text used to describe the filtering process highlights that the most commonly used terms were “splice,” “missense,” “protein,” “frameshift,” “stop gain,” “loss of function” (LoF), and “protein-truncating variant” (PTV), but a wide range of terms were used (Figure S5). Variants were often filtered by both annotation/predicted effect and MAF thresholds, with multiple different filtering criteria used per publication (examples are provided in Table S3).

The number of variants analyzed in WES single-variant analyses is considerably less than those typically analyzed in array-based GWASs (median, 158,091; versus 5,554,549), whereas, in WGS single-variant analyses, the number is greater (median, 12,210,410) (Table 1). The median number of statistical tests performed in aggregate analyses was 18,360, approximating the number of protein-coding genes with a consensus CDS (19,033; coding DNA sequence)^20^ because the most-common unit over which variants are aggregated is the protein-coding gene. The analyses in which the number of tests was greater than the inter-quartile range were those in which the unit of analysis was non-genic. The most-common non-genic aggregation units we observed were regulatory regions^18,19,21,22^ or agnostic sliding windows.^23–26^ Authors also aggregated across evolutionary conserved regions or pathways.^19,27^

The outcome of the various variant filters or “masks,” i.e., a list of the qualifying variants included in each analysis, was not provided in any of the 167 publications we analyzed. However, some publications did specify the number of qualifying variants included per unit of aggregation.^28,29^

### Sample characteristics

We next surveyed the characteristics of samples (sample size, ancestry, and traits) studied in seqGWAS. We compared the sample sizes of the seqGWAS, because that is a key determinant of statistical power. We classified publications into bins based on the number of individuals in the publication (Figure S6). The most-common sample size bin was 300–3,000 individuals (43% of publications), but in the past few years, there has been a near-even distribution across bins from small to large sample sizes. In 2019, both the smallest (<300 individuals) and the largest (>10,000) sample-size bins were used in approximately a quarter of publications each (23% and 26%, respectively; Figure S6). The number of cases is also a component of statistical power, and unbalanced case/control ratios can inflate type 1 errors.^30^ We observed 10 publications (6%) with unbalanced case/control ratios (cases ≤ 15% of samples), most of those (n = 7, 4%) being highly unbalanced (cases ≤ 4% of samples) (Table S4).^31–33^

**Figure 3.**
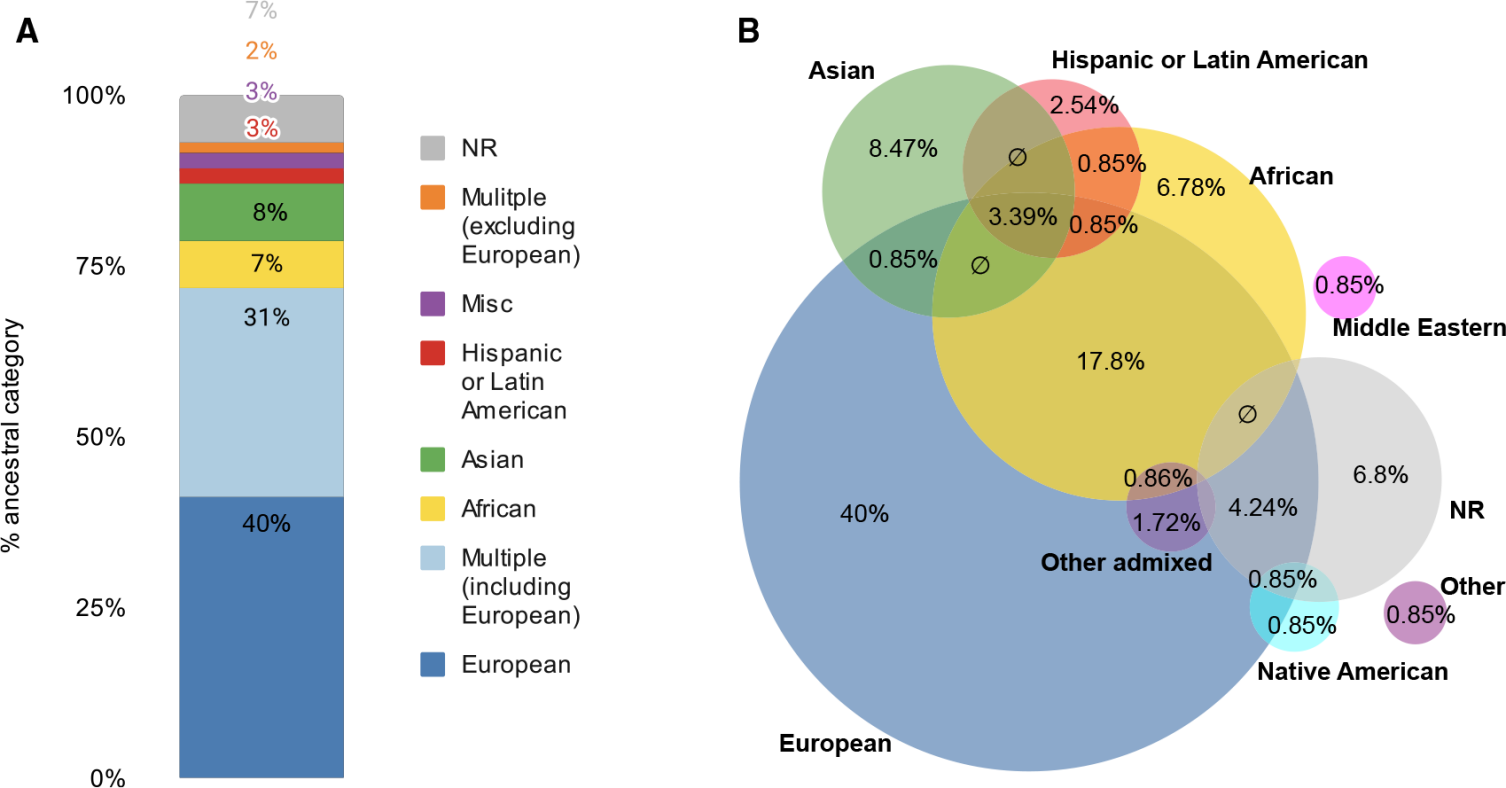
Ancestry of individuals used in sequencing-based GWAS publications Publication-level breakdown of the broad ancestry categories, defined per the GWAS Catalog ancestry framework.^36^ Some categories are collapsed for ease of display, analysis is based on 2014–2019 publications, n = 120. (A) Overview of the percentage of publications that included only one or multiple ancestral categories. (B) The proportion of publications that included the specified broad ancestral category. Overlaps indicate multiple ancestries were included in one publication; indicates an empty set. Venn diagram was created using DeepVenn.^38^ Note that Venn diagrams of this size cannot be fully proportional (see Figure S7 and Table S5 for full data).

The inclusion of diverse ancestral backgrounds in genomics studies is recognized as important,^34,35^ but analysis of array-based GWASs has highlighted the extreme bias toward samples of European origin.^36,37^ We assessed and compared ancestry in seqGWAS. Following the GWAS Catalog ancestry framework (a standard methodology for representing ancestry),^36^ we extracted publication-level, broad ancestral categories of samples. Mirroring what has been seen elsewhere with array-based GWASs, 71% of all publications (n = 85/120) included European ancestry individuals, with 40% not including any other ancestry (n = 48/120) (Figure 3A; Table S5). The second most commonly examined ancestral group was African American (28% of publications, n = 33/120), and most of those publications (21%) also included other ancestries (Figures 3B and S7). This profile may, in part, be due to the presence of large, trans-ancestry consortia, such as the Trans-Omics for Precision Medicine (TOPMed) program, which is the most commonly occurring consortium or cohort mentioned (Table S7).

We also examined the number of traits analyzed within the reported association study. Most publications examined one or two traits (76%, n = 89), whereas a few (4%, n = 5) examined 55–75 traits as part of larger-scale studies.^18,22,39–41^ More recently (2019–2020), very-large-scale studies using the UK Biobank have included 791–4,262 traits^42–44^ (Figure S8). Non-UK-Biobank publications analyzing multiple traits were mostly focused on quantitative biomarker or metabolite-level-type traits,^18,21,41,45^ such as inflammatory biomarkers, blood metabolite levels, blood protein levels. Studies analyzing fewer traits were more likely to be case/control studies.^46–49^ A full list of publication-level trait names (analogous to the GWAS Catalog “reported trait”) and corresponding mapped Experimental Factor Ontology (EFO) terms are provided in Table S4.

### Data availability

The public availability of full summary statistics from GWASs has great potential to extend the power of initial studies by enabling the community to re-analyze, meta-analyze, and perform follow-up analyses, with minimal risk to participants.^11,50^ We assessed whether summary statistics, in addition to individual-level genotyping results, were reported in these publications as available without restriction in a public repository. Sharing of sequencing-based single-variant summary statistics was much lower (5% of publications, n = 4/79, 2014–2019) than the proportion of array-based publications in the GWAS Catalog in the same period (12% of publications, n = 300/2,571, 2014–2019) (Table 1). Sharing of array-GWAS summary statistics is greater in recent years (19% of 2019 GWAS Catalog publications, n = 101/527), but seqGWAS summary statistics still lag (9%, n = 3/32). A further 2.5% of sequencing publications (n = 3/120, 2014–2019) deposited summary statistics in a controlled-access public repository (the Database of Genotypes and Phenotypes [dbGAP]). In contrast, 24% of publications (n = 29/120) deposited individual-level sequencing data in controlled access repositories (dbGAP or European Genome-Phenome Archive [EGA]) (Table S6) and, for some summary-level data, may have been co-submitted or bundled with those data but not specifically stated by the authors.

The data content of single-variant summary statistics for seqGWAS is comparable with that for standard-array GWASs and can conform to emerging standards.^11,50^ However, summary statistics for aggregate analysis in seqGWAS are commonly composed only of a gene name (or other range specifying chromosomal coordinates), p value, and often the number of contributing variants, sometimes separated by cases/controls. Crucially, we did not observe any publications that reported the list of variants included in each aggregate unit, which is key to interpretation of the data, either in the main text or in accompanying material.

## DISCUSSION

### Recommended standards

Based on our review and analyses, we recommend standards to improve the reporting and accessibility of seqGWAS. First, to increase transparency when referring to study design and facilitate identification, we recommend that the community adopt the name of “sequencing-based GWAS,” abbreviated as “seqGWAS” (Box 1, recommendation 1). Second, to enable accurate interpretation and comparison of results across studies and loci, it is essential that detailed information describing each association test (including statistical tests and contributing variants) are consistently reported (Box 1, recommendations 2 and 3). These recommendations are based upon, and are designed to address, our observations of the state of the field.

### Observations

The sequencing-based association studies in the publications we analyzed included either single or aggregate multi-variant analyses. The restriction of single-variant analyses to common variants renders those studies largely comparable with array-based GWASs (Figure 2), with similar implications for data content and reporting (Box 1, recommendation 2) and similar utility for re-use, for example, in the derivation of polygenic scores or in Mendelian randomization. In comparison, studies performing tests of aggregate association across multiple variants, which appear in most (81%) publications, focus on “low-frequency,” “rare,” and “ultra-rare” variants. Multiple statistical models of aggregate association are frequently used in the same publication because the power of each test depends on how closely the assumptions of the model match the true disease etiology at each locus. Therefore, there is no best model (including statistical tests and variant filtering strategies) across loci and traits, and there is no best model necessarily knowable *a priori*. To enable accurate interpretation and comparison of results across studies and loci, it is, therefore, essential that detailed information describing each association test (including statistical tests and contributing variants) is consistently reported (Box 1, recommendations 2 and 3).

It is in the performance and, therefore, reporting of aggregate association tests that sequencing-based association studies differ most from standard array-based GWASs. We observed that the experimental information provided for aggregate tests was not sufficient to facilitate thorough examination or replication. Variants are filtered (typically by MAF and functional annotation/predicted consequence) and combined in different units of aggregation. Crucially, the list of variants contributing to each test is not provided by these publications. Availability of these data would facilitate attempts at replication and enable further analysis and functional investigation^51^ (Box 1, recommendation 3b).

Given the rarity of these variants, privacy concerns regarding de-identification may be a barrier to their sharing. We suggest that the community look to the field of rare-variant clinical genomics, in which it is becoming increasingly accepted that the potential benefits of sharing far outweigh the perceived risks.^52^ This is illustrated by the number of clinical-laboratory-derived variants in ClinVar more than doubling since 2018.^53,54^ We note that individual genetic variants, even very rare ones, are not uniquely identifying and would require in-depth knowledge of an individual’s genotype to connect an individual to a phenotype.

Theoretically, lists of qualifying variants could be recapitulated, but filtering information provided by authors is again diverse and often vague and, overall, insufficient to independently derive those lists. The community should consider standardized ways to communicate variant filters or masks (for example, using the sequence ontology to describe functional annotation/predicted functional effect filters^55^). The unit of aggregation, which encompasses the variants included in each test (typically gene), must be clearly defined. This should include the coordinates of the region and the genome assembly or annotation release, along with any additional variant-filtering information (Box 1, recommendation 3a).

We observed that a smaller proportion of full-summary statistics are publicly available from seqGWAS (5%) compared with array-based GWASs (12%). That percentage is low for both types of studies despite guidance and growing community consensus supporting sharing (web resources).^50^ There are a number of reasons why full and public data sharing may be less for sequencing than array-based studies. There may be additional perceived privacy concerns regarding the rare variants present in sequencing-based summary statistics. It is also possible that summary statistics may be bundled with the individual-level genotyping data that 24% of publications deposited in controlled-access repositories (dbGAP/EGA). Single-variant summary statistics can conform to the proposed array-based standards (Box 1, recommendation 2)^11^ and can already be submitted to the GWAS Catalog. However, aggregate-analysis summary statistics, when they are shared, are typically only a gene name and a p value (sometimes with the number of qualifying variants included). These files are not large or cumbersome, given that the number of human genes is only approximately 20,000 and are easy to share, for example, as a supplementary table. As described above, we recommend authors supply full lists of qualifying variants that contribute to each test (Box 1, recommendation 3b). We hope that the development and adoption of these standards will simplify and encourage the sharing of seqGWAS summary statistics.

The ability of sequencing to genotype all variants present in the cohort offers a significant opportunity to overcome the biases inherent in array-based genotyping, with the potential to reduce disparities among ancestry groups. Despite that, the bias toward European-ancestry populations observed in array-based GWASs (49% European only and 74% including European) remains in sequencing publications (40% European only and 71% including European). Furthermore, we note that the percentage of European sequencing-based analyses is likely to be greater; publications containing multiple GWASs are more likely to be from large cohorts with deep phenotyping data, which are predominantly European (e.g., UK Biobank). Given the advantages of sequencing in analyzing non-Europeans, we question why it is not being further used. There are many possible reasons for this, including increased cost, the lack of diversity in legacy cohorts, pre-existing consent agreements, privacy concerns associated with rare-variant analysis, and analysis methods being complex. The GWAS Catalog reiterates its stance in encouraging analysis of diverse populations and encourages researchers to take advantage of the opportunities offered by sequencing technologies in enabling unbiased genotyping across ancestries (Box 1, recommendation 4).

### Limitations of the study

The lack of standardized terms to refer to seqGWAS creates challenges for the reliable identification of these publications using term-based literature-search methods. The 167 publications we identified are, therefore, certainly an underestimate of the number of publications, and we do not claim that this work is a comprehensive analysis of all published seqGWAS. To maintain consistency and enable comparability across studies, we decided to limit our analysis to publications carrying out an unbiased, genome-wide or exome-wide assessment of loci associated with traits, equivalent to the GWAS Catalog’s inclusion criteria (web resources). Many of the publications we screened and deemed ineligible were targeted analyses based on prior knowledge, for example, to specific loci, genes, or pathways and are scientifically valid studies but are out of the scope of this manuscript. In our recommendation of the term “seqGWAS” (Box 1, recommendation 1), we note that some may feel the use of “GWAS” is inappropriate, primarily because WES-based analyses are necessarily targeted to expressed regions. However, we observe that the term “GWAS” is commonly used to refer to both genome-wide and exome-wide array-based association studies. Our motivation for suggesting a unique nomenclature (sequencing-based GWAS/seqGWAS) is to facilitate the “find-ability” of these study types (large-scale association studies that analyze variants spread across the genome (e.g., with coverage across all autosomal chromosomes) in the scientific literature.

A necessary limitation of this work is its restriction to a specific time period (2014–2020), and as such, it serves as a snapshot of the state of the field. It is anticipated that the field will grow significantly in the immediate future, and the ratio of WES and WGS studies may change. However, the findings of our work, in terms of how studies are described and reported, are unaffected by whether or not they are WES or WGS or the total number of studies. The recommendations similarly apply to both coverage types. Furthermore, we believe this is an appropriate time to publish a study such as ours so that standards can be established sooner, thus enabling future publications to adhere to the FAIR principles.

### Ensuring seqGWAS are FAIR

The maximum benefit of scientific research can only be realized if data are FAIR (findable, accessible, interoperable, and reusable), as described by the FAIR guiding principles for good scientific data management.^13^ Our analysis highlights several obstacles to implementation of these principles for seqGWAS, including lack of an appropriate resource or repository to store and disseminate the data, consistency of metadata reporting without the use of structured vocabularies, clarity on metadata indexing that needs to support searching, and a community standard for summary statistics. The GWAS Catalog’s primary aim is to provide a comprehensive resource and repository of all large-scale genomic association studies and, as such, has extended its scope to include seqGWAS, initially focusing on single-variant analyses. We will support the community to reach consensus on the reporting of aggregate seqGWAS, including the creation of standards for metadata and summary format and content.^50^ The development and adoption of reporting standards will increase the availability, accessibility, and utility of seqGWAS. We include a summary of our recommendations (Box 1) and welcome further input from the community.

## STAR★METHODS

Detailed methods are provided in the online version of this paper and include the following:

### RESOURCE AVAILABILITY

#### Lead contact

Further information and requests for resources and reagents should be directed to and will be fulfilled by the lead contact, Aoife McMahon (aoifem@ebi.ac.uk).

#### Materials availability

This study did not generate new unique reagents.

#### Data and code availability

Data underlying analyses in this paper are curated from the literature and are presented in Table S4.

This paper does not report original code.

Any additional information required to reanalyze the data reported in this paper is available from the lead contact upon request.

### METHOD DETAILS

To enable direct comparability with array-based GWAS we defined sequencing-based association studies as studies that analyze associations between a trait and a genome-wide distribution of genetic variants from either whole-genome or whole-exome sequencing. This does not include targeted sequencing studies that are limited to specific genomic regions or subsets of genes (e.g., publications^57–59^). From these, we selected studies with population-based association analyses, and did not include studies that used family structure/linkage (e.g., publications^60–62^) or were aimed at diagnostic discovery of pathogenic variants (e.g publications^63–65^). We also included family-based association studies, but only if they performed standard association analysis with relatedness accounted for in the model (e.g., publications^39,66^). Studies that combine array and sequencing-based genotyping, such as partially array-genotyped, or array genotyped with sequencing data used as an imputation panel, were not included in our analyses.

Sequencing-based association publications meeting these inclusion criteria were identified by several routes: Pubmed and EuropePMC literature searches, the GWAS Catalog machine learning-based literature search,^56^ examination of grants, cohort and project websites, social media, conference talks, references in publications and personal communications (Table S1). The source of initial identification of each sequencing publication was recorded. Publication level metadata relating to study design, sample description, traits examined and data availability were extracted (Tables S2 and S4). Publication triage, eligibility assessment and extraction of metadata were performed by experienced GWAS Catalog curators. Analysis of study eligibility, genomic coverage and analysis type was performed for 2020 publications. More detailed analysis of the sample, trait, data sharing and statistical tests was available to the end of 2019.

### QUANTIFICATION AND STATISTICAL ANALYSIS

For analysis of text related to variant types, curators extracted sentences describing variant selection and relevant terms were identified using the text analysis tool MonkeyLearn (https://monkeylearn.com/word-cloud/). The output was examined by expert curators and non-relevant terms excluded, terms collapsed and missed relevant terms were added and counted.

## Supplementary Material

1

2

## Figures and Tables

**Table 1.** Availability of summary statistics and number of statistical tests performed in sequencing versus array-based GWASs

	Single-variant array, % (n)	Single-variant sequencing, % (n)	Aggregate sequencing, % (n)

Summary statistics available without restriction	12 (300)	5 (4)	7 (7)

Number of tests (reporting)			

Reported	91 (5,817)	74 (61)	81 (84)
Not reported	9 (610)	26 (21)	19 (20)

Number of tests (distribution)	overall	overall	overall

Minimum	12,033	26,011	339
Q1	899,892	144,477	16,788
Median	5,554,549	548,889	18,665
Q3	9,334,585	8,752,596	20,843
Maximum	90,000,000	32,503,121	129,820,320

		WES only	WES only

Minimum	–	26,011	735
Q1	–	81,843	16,751
Median	–	158,091	18,360
Q3	–	235,133	20,000
Maximum	–	1,810,198	88,183

		WGS only	WGS only

Minimum	–	658,234	339
Q1	–	7,666,134	19,903
Median	–	12,210,410	32,316
Q3	–	29,880,479	1,082,577
Maximum	–	32,503,121	129,820,320

Publications that state that they share summary statistics openly (not including those provided with restricted access). Reported/not reported refers to whether the number of statistical tests performed was detailed in the publication. The number of statistical tests performed in sequencing-based studies is based on publications that provide one “number of statistical tests” (n = 51 of 79 for single-variant analysis, n = 56 of 101 for aggregate analysis). Publications that provide a range of statistical test numbers performed are included in the “reported” category but are not included in the distribution. The data for array-based GWAS were obtained from 2014–2019 studies in the GWAS Catalog (December 2, 2020 release) (see STAR Methods).

**KEY RESOURCES TABLE:** 

REAGENT or RESOURCE	SOURCE	IDENTIFIER

Software and algorithms		

Text analysis tool (MonkeyLearn)		https://monkeylearn.com/word-cloud/
GWAS Catalog machine learning-based literature search	Lee et al.^56^	N/A
Literature search engine, EuropePMC		http://europepmc.org
PubMed		https://pubmed.ncbi.nlm.nih.gov

Other		

Literature (primary research journal articles)	Peer reviewed journals	PubMed IDs listed in Table S4
Publicly available curated meta-data	NHGRI-EBI GWAS Catalog	https://www.ebi.ac.uk/gwas/

## Box 1. Recommendations for sequencing-based GWAS reporting standards

Our recommendations for the development and adoption of reporting standards to increase the availability, accessibility, and utility of sequencing-based GWASs. The GWAS Catalog will support deposition of these datasets and promote adoption of these standards as well as continued discussions to reach consensus on the reporting of aggregate analyses.

1. WGS and WES association studies be referred to as “sequencing-based GWASs” (seqGWAS)
2. Single-variant analysis summary statistics be
  - Reported using the same standards as proposed for single-variant array-based GWASs^11,50^
  - Shared openly by submission to the GWAS Catalog
3. Aggregate analyses:
  - Metadata be reported to enable interpretation and aid reproducibility including
    - Sufficient details of the statistical test to allow replication of results
    - Minor allele frequency thresholds used
    - Details of tools used for functional annotation/consequence prediction (e.g., VEP release 103) and ontology terms used to describe the consequence (e.g., Sequence Ontology)
  - Community reaches consensus for standard content and format for reporting of aggregate seqGWAS summary statistics. This should include
    - The full list of qualifying variants contributing to each test
    - Chromosomal coordinates of aggregation units (including genome assembly builds or gene annotation release version, e.g., GENCODE release 37, GRCh38)
    - A standard identifier for the aggregation unit, e.g., HGNC gene name or symbol (if applicable)
    - p value
4. SeqGWAS studies be conducted in populations that include more diverse ancestries

## Highlights

- Recommendations for increasing FAIRness of sequencing-based GWASs
- To be findable, we recommend standard terminology of sequencing-based GWAS (seqGWAS)
- To improve access and standards, the GWAS Catalog will support deposition of seqGWAS
- To improve utility, we recommend reporting standards for single and aggregate analyses
