# Supplementary material for: Sequencing-based genome-wide association studies reporting standards
Source: Cell Genom. 2021 Oct 13;1(1):100005. doi: 10.1016/j.xgen.2021.100005 (PMC8637874; doi:10.1016/j.xgen.2021.100005)
Supplement: Document S1. Figures S1–S8 and Tables S1–S3 and S5–S6 [file mmc1.pdf]

Cell Genomics, Volume *1*

Supplemental information

Sequencing-based genome-wide

association studies reporting standards

Aoife McMahon, Elizabeth Lewis, Annalisa Buniello, Maria Cerezo, Peggy Hall, Elliot Sollis, Helen Parkinson, Lucia A. Hindorff, Laura W. Harris, and Jacqueline A.L. MacArthur

Supplemental Figures

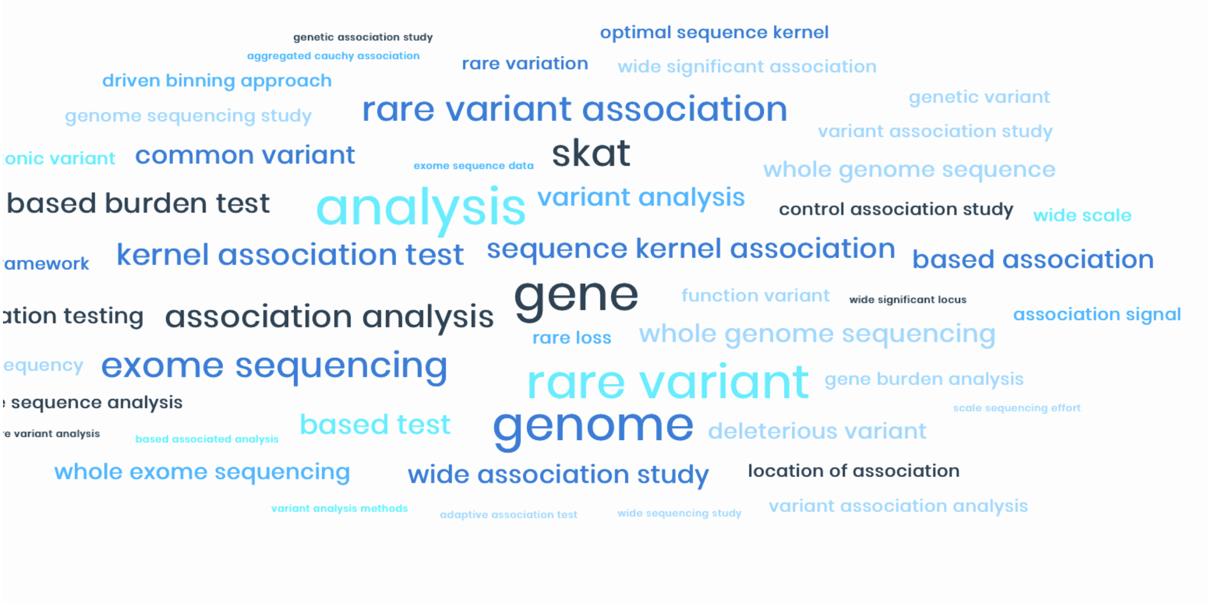

Supplemental Figure 1
Word cloud from abstract and title text related to the study design of publications (including aggregate and single analysis) (related to Figure 1).
Word size corresponds to frequency x relevance metric (inverse document frequency of the term in an unrelated corpus of text), the top 50 enriched terms are displayed.

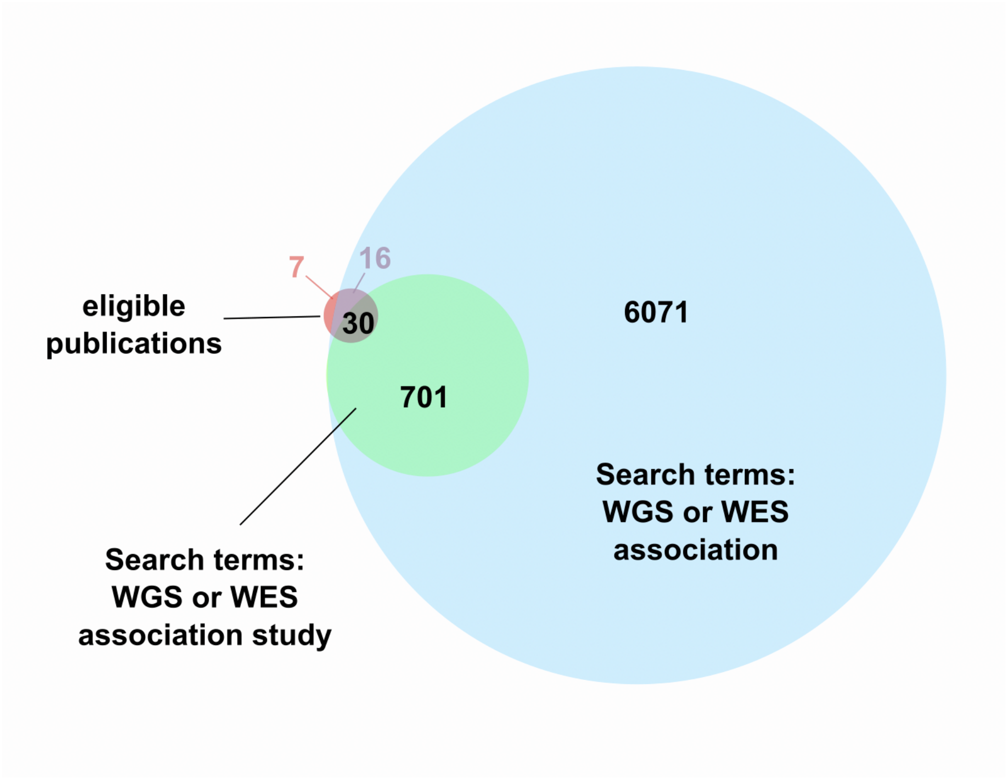

Supplemental Figure 2

An illustration of the difficulty in ascertainment of sequencing-based GWAS publications (Related to STAR methods).

The overlap between the list of eligible 2019 publications with the search results of permissive query searches conducted using a literature search engine (EuropePMC). This analysis is limited to 2019. The labels shown on the diagram represent specific search terms used in EuropePMC. Label (WGS or WES association) = Query (("WGS" AND "association") OR ("whole genome sequencing" AND "association") OR ("WES" AND "association") OR ("whole exome sequencing" AND "association")) AND (FIRST_PDATE:[2019-01-01 TO 2019-12-31])

Label (WGS or WES association study) = Query (("WGS" AND "association study") OR ("whole genome sequencing" AND "association study") OR ("WES" AND "association study") OR ("whole exome sequencing" AND "association study")) AND (FIRST_PDATE:[2019-01-01 TO 2019-12-31])

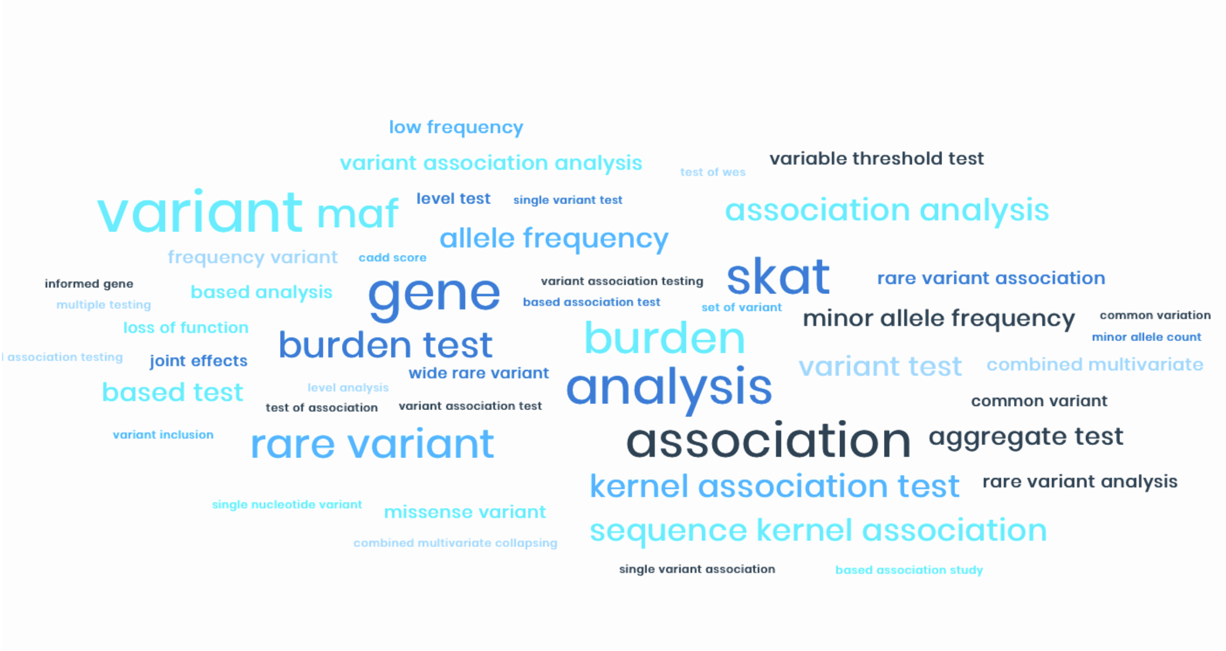

Supplemental Figure 3

Word cloud from text related to the study design of publications which perform aggregate analysis, from sections other than abstract and title (related to Figure 2). Word size corresponds to frequency x relevance metric (frequency-inverse document frequency (TD-IDF), the top 50 enriched terms displayed.

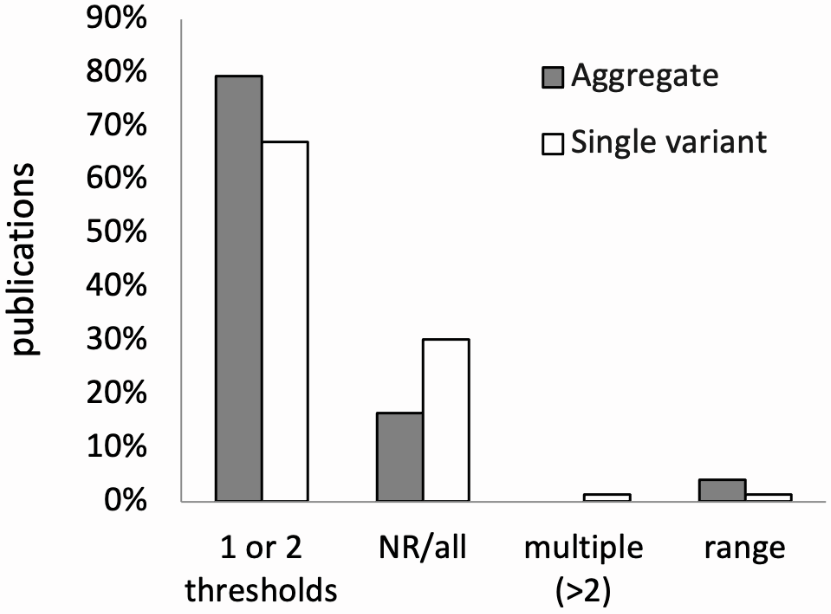

Supplemental Figure 4
Reporting of minor allele frequency thresholds in single variant and aggregate analyses (related to Figure 2).
This figure shows how MAF was reported in publications (2014-2019) (single variant: n publications = 97, aggregate: n publications = 76). Data in Figure 2 are derived from those publications that report one or two thresholds. 'NR/all' includes publications that provided no information on thresholds as well as publications which implied that all variants were included.' 'Range' represents publications that included variants within a specific range of MAFs e.g. 1-5%. NR = not reported.

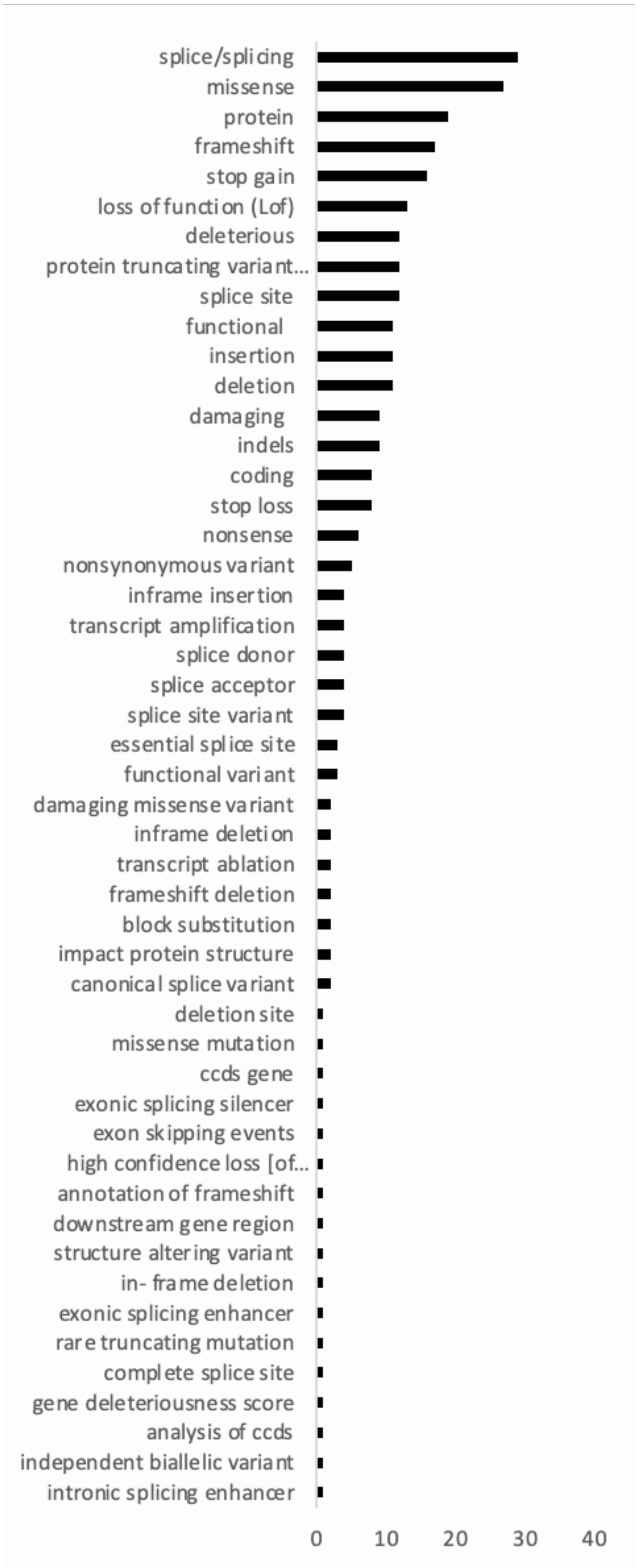

Occurrences of terms in text related to variant selection

Supplemental Figure 5
Language describing variant types (related to Figure 2)

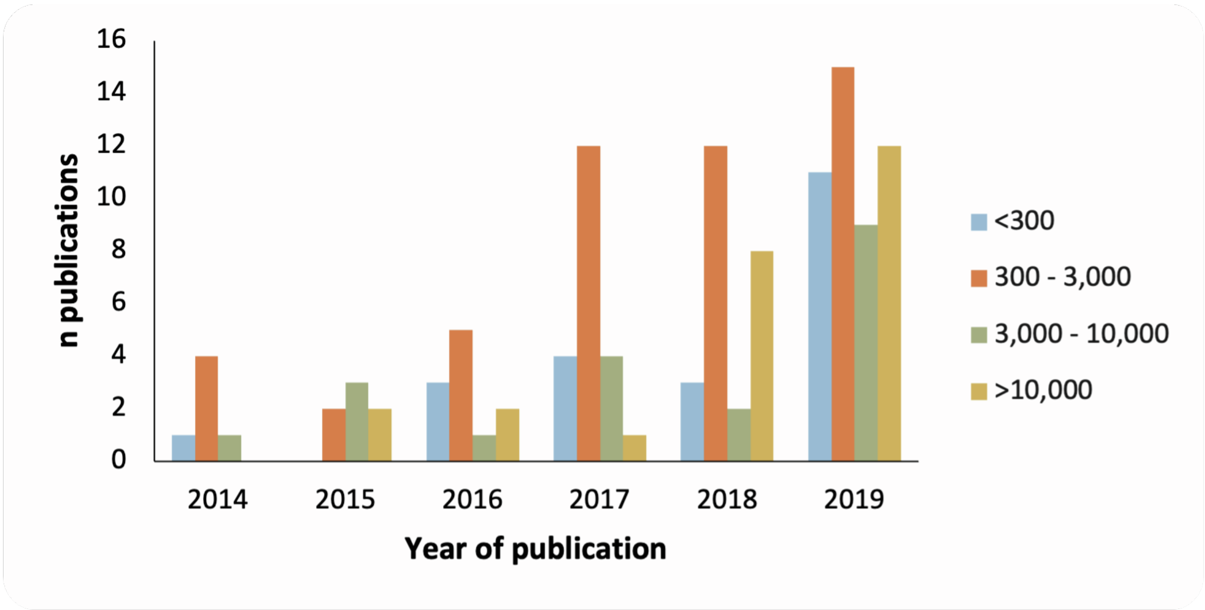

Supplemental Figure 6
Sample size bins in sequencing-based association studies (related to Figure 3).
Publication level sample sizes were classified into brackets of <300, 300-3000, 3000-10,000 or >10,000 individuals. Number of publications in each sample size bracket, by year.

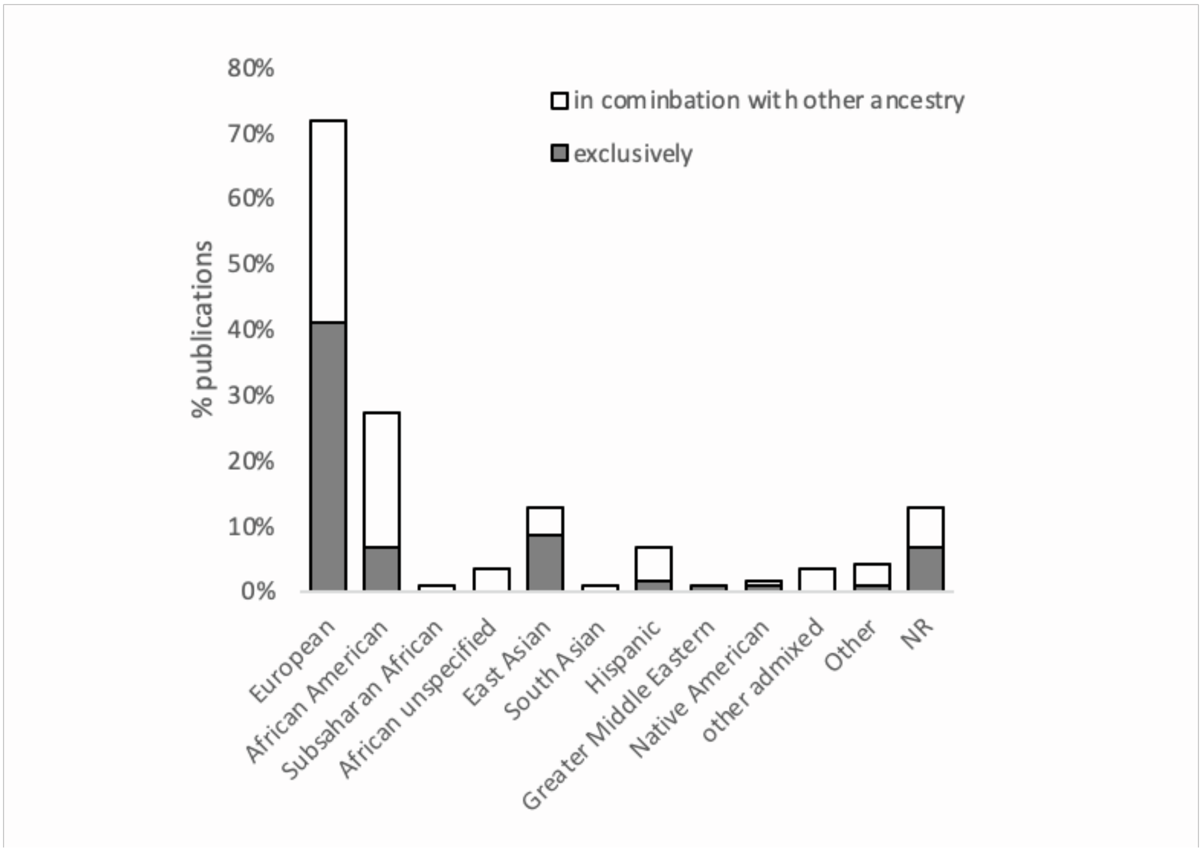

Supplemental Figure 7
Detail on percentage of publications including ancestries exclusively or in combination with other ancestries (related to Figure 3).

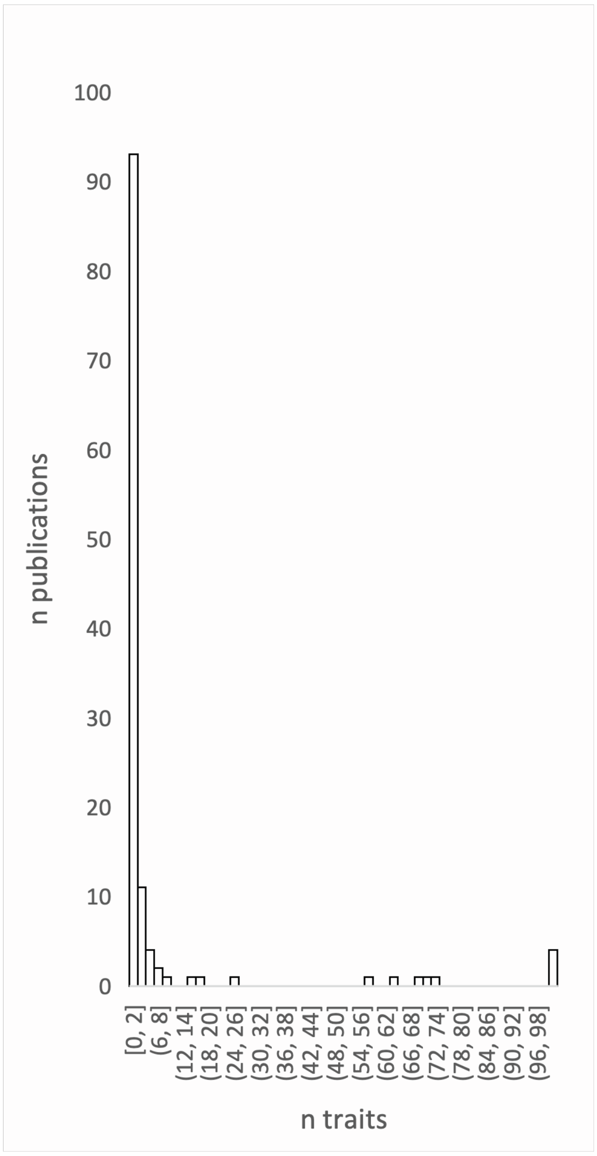

Supplemental Figure 8

Number of traits analysed in sequence-based association publications (related to STAR Methods).

Distribution of number of traits analysed per publication. The final overflow bar represents >100 traits (publications of 644, 791 and 2048 traits).

SUPPLEMENTAL TABLES

Publication identification sources
**GWAS Catalog machine learning search:**
see Lee et al[3]
**Pubmed and EuropePMC text query searches:**
sequencing association genome OR exome
sequencing association genetic
rare variant whole genome whole exome
rare variant association analysis
gwas sequencing population
genetic association studies"[MeSH Terms] AND "high-throughput nucleotide sequencing"[MeSH Terms] NOT Review[ptyp]
("whole genome sequencing" OR "whole exome sequencing") AND (METHODS:"skat-o" OR METHODS:"gene-based" OR METHODS:"single variant" OR METHODS:"burden")
("whole exome sequencing"[MeSH Terms] OR ("whole"[All Fields] AND "exome"[All Fields] AND "sequencing"[All Fields]) OR "whole exome sequencing"[All Fields]) AND ("association"[MeSH Terms] OR "association"[All Fields])
("rare variant association" AND "whole genome sequencing" OR "whole exome sequencing")
("gene-based" OR "collapsing analysis" AND "whole exome sequencing" OR "whole genome sequencing")
"skat-o" AND "sequencing"
"rare variant" sequencing association whole-genome
**Cohort/project website:**
TopMEd publications list (https://www.nhlbiwgs.org/publications)
NIH project reporter (https://projectreporter.nih.gov)
UKBiobank list (in house)
Open Targets list (in house)
**References**
**Twitter**
**Conferences**
**GWAS Catalog author summary statistics submission**
**Personal communication**

Supplemental Table 1
Sources where sequencing-based GWAS were identified (related to STAR Methods)

	Curated meta-data	2014-2019	2020 + 2019/2020 preprints
	n:	120	40 + 7
**Study design:**	*Coverage (WGS/WES)*	✓	✓
	Analysis type (single/aggregate)	✓	✓
	Number of statistical tests (range or number)	✓	✗
	Minor allele frequency thresholds (if >2 provided extracted as 'multiple', if 2 extracted both)	✓	✗
	Reference genome	✓	✗
	Terms related to study design (abstract/title, elsewhere)	✓	✗
	Terms related to qualifying variants	✓	✗
**Sample:**	*Sample size category (<300=0, <3000=1, <10,000=2, >10,000=3)*	✓	✗
	Broad ancestral category	✓	✗
	Additional ancestry descriptor	✓	✗
	Country of recruitment (if ancestral category NR)	✓	✗
	Consortium/Cohort	✓	✗
**Traits:**	*Number analysed*	✓	✗
	Reported trait	✓	✗
	Mapped trait EFO name	✓	✗
	Mapped trait EFO ID	✓	✗
	Background trait EFO name	✓	✗
	Background trait EFO ID	✓	✗
**Data availability:**	**Summary statistics**		
	Single variant/aggregate; freely, restricted, partial or no.	✓	✗
	Location	✓	✗
	**Sequence data**		
	In restricted repository or no	✓	✗
	Location	✓	✗
	Accession ID	✓	✗

Supplemental Table 2 Overview of publication meta-data extracted (related to STAR Methods). All curated meta-data is included in Supp. Table 4.

**Selected examples of variant filtering descriptions (annotation/function)**
non-synonymous
putative damaging
nonsynonymous and otherwise presumed functional
variants that are most likely to affect a protein's function, that is, non-synonymous, stop gain, stop loss, frameshift deletions and insertions, and splice site variants.;
nonsynonymous and splice-site variants
“qualifying” variants; 1) all non-synonymous and canonical splice variants (coding model), 2) all non- synonymous coding variants except those predicted by PolyPhen-2 HumVar(13) to be benign (not benign model), and 3) only stop gain, frameshift and canonical splice variants (loss-of-function [LoF] model).
All aggregation tests utilized only variants that were rare (defined as MAF<5% in the population set) and either truncating (frameshift, essential splice site, nonsense) or missense and predicted to be deleterious (by at least one of Polyphen, SIFT, or Condel) as annotated by Variant Effect Predictor (VEP) release 74. The analysis of rare truncating mutations, however, only included variants annotated as nonsense (SNVs only), essential splice site (SNVs/indels), or frameshift (indels only). (multi)
ultrarare, deleterious, nonsynonymous variants ; qualifying variants were restricted to indels and single-nucleotide variants annotated as having either a loss-of-function (LoF) effect, an in-frame indel, or a “probably damaging” missense prediction by Polymorphism Phenotyping version 2 (PolyPhen, HumDiv; http://genetics.bwh.harvard.edu/pph2/) (16). These analyses relied on the predicted effects of the LoF and missense annotated variants whose functions have not been individually confirmed in the laboratory. We subsequently performed analyses of CCDS genes using six alternative qualifying variant models as defined in Table E4, including an autosomal recessive model and a synonymous variant negative control model.
We defined qualifying variants in four ways (Table 1); ultra-rare variants; loss-of-function, inframe insertion or deletion, or a “probably damaging” missense effect by PolyPhen-2 (HumDiv); Three secondary analyses were performed to evaluate the contribution to epilepsy risk from: rare loss-of-function variants with an internal and external population MAF up to 0.1%; rare non-synonymous variation in the general population with an internal and external MAF up to 0.1%; and a presumed neutral model that imposed similar MAF thresholds as our primary analysis, but focused specifically on protein-coding variants predicted to have a synonymous effect.
deleterious - predicted by variant effect predictor (VEP) to have “HIGH” impact, cause protein loss-of-function (stop-gain, frameshift insertion and deletion [indel], etc.), or were missense mutations with a combined annotation dependent depletion (CADD)26 score >25
we considered six functional annotations, CADD [7], RegulomeDB [18], FunSeq [19], Funseq2 [20], GERP++ [21] and GenoSkyline [8]
loss of function (LoF) variants defined as follows were used for further analysis: stop gain/loss, coding INDELs, splice-site acceptors, and splice-site donors. We also included variants predicted as damaging according to their SIFT [23] score and a CAD [24] score of > 20.; gene score (a gene deleteriousness score) quantified the impact of damage of a gene, and was defined as the geometric mean of the SIFT scores for the multitude of deleterious variants in a gene.
Two sets of analyses were performed: The first included only frameshift (insertion/deletion/block substitution), stopgain, stoploss and splicing SNVs (jointly defined as loss-of-function (LOF) variants), while the sec- ond included all variants captured in the first analysis as well as non-synonymous SNVs and non-frameshift indels or block substitutions that were predicted to be probably dam- aging by Polyphen 2 and deleterious by SIFT [1, 62].
(1) PTVs at any allele frequency with VEP annotations: frameshift_variant, initiator_codon_variant, splice_acceptor_variant, splice_donor_variant, stop_lost, stop_gained;<br>(2) PTVs included in (1) plus missense variants with MAF<0.1% scored as “damaging” or “deleterious” by all five functional prediction algorithms;<br>(3) PTVs included in (1) plus missense variants with MAF<0.5% scored as “damaging” or “deleterious” by all five algorithms. (multi)

Supplemental Table 3

Examples of variant filtering descriptions provided by authors (related to Figure 2).

Terms in text related to variant selection.

Supplemental Table 4
Full curated meta-data from publications included in this analysis

Supplied as separate .xlsx file

Broad ancestral category	Overall % (n)		Exclusively % (n)		In combination with other ancestry % (n)	
European	71%	(85)	40%	(48)	31%	(37)
African American	28%	(33)	7%	(8)	21%	(25)
Subsaharan African	1%	(1)	0%	(0)	1%	(1)
African unspecified	3%	(4)	0%	(0)	3%	(4)
East Asian	13%	(15)	8%	(10)	4%	(5)
South Asian	1%	(1)	0%	(0)	1%	(1)
Hispanic	8%	(9)	3%	(3)	5%	(6)
Greater Middle Eastern	1%	(1)	1%	(1)	0%	(0)
Native American	2%	(2)	1%	(1)	1%	(1)
Other admixed	3%	(4)	0%	(0)	3%	(4)
Other	4%	(5)	1%	(1)	3%	(4)
NR	13%	(15)	7%	(8)	6%	(7)

Supplemental Table 5
Publication level breakdown of the broad ancestral categories of individuals, defined per the GWAS Catalog ancestry framework (related to Figure 3).
Overall = percentage of all publications that include an ancestry, either exclusively or in combination with other ancestries.

**Individual level sequence data availability**	%	N publications
Controlled access repository (accession ID provided)	19%	23
Controlled access repository (no ID provided)	2%	2
Partial dataset in repository	2%	2
Partial dataset in repository, partial available upon request	2%	2
Available upon request	3%	4
None	73%	90

Supplemental Table 6
Individual level sequence data availability (related to Table 1).
Analysis of author statements regarding individual level sequence data.

Cohort/consortium	Count
NR	24
TOPMed	15
ARIC	8
NHLBI GO ESP	8
JHS	6
Alzheimer Disease Sequencing Project (ADSP)	6
TwinsUK	6
UK10K	5
UKBiobank	5
FHS	4
FINRISK	3
ADNI	2
CHARGE	2
Estonian Biobank	2
GenTAC	2
HELIC-MANOLIS	2
IGM	2
Epi4K	2
Old Order Amish Study	2
ALSPAC	1
ARC	1
ARRA	1
AURORA	1
BDR	1
Boston Early-Onset COPD Study (EOCOPD)	1
CASPMI	1
CHS	1
CONVERGE	1
COPDGene	1
CUMC	1
deCODE	1
DiscovEHR	1
EGD	1
Emory	1
ENGAGE	1
EPGP	1
EPIC Potsdam	1
Epilepsy Phenome/Genome Project	1
Familial dyslipidemia	1
FinMetSeq	1
FinnDiane	1
Genetic Epidemiology of Asthma in Costa Rica	1
Genomic Translation for ALS Care (GTAC study)	1
Georgia Centenarian Study (GCS)	1
GOLDN	1
Health 2000	1
Healthy Nevada Project	1
iJGVD (controls)	1
International FTLD-TDP WGS Consortium	1
INTERVAL	1
IRASFS	1

IRCCS	1
KARE	1
MESA	1
METSIM	1
Minnesota Twin Family Study (MTFS)	1
Nottingham Smokers cohort	1
NSPHS	1
OPCS	1
PACA-AU	1
PAH biobank	1
PanCuRx	1
PDAY	1
PEACH	1
PREDICTION-ADR Consortium and EUDRAGENE	1
PROP	1
QPCS	1
RISK	1
ROSMAP	1
RS	1
SABG	1
SDR	1
SJLIFE	1
Steno Diabetes Center	1
T2D-GENES	1
TCGA	1

Supplemental Table 7
A count of the occurrence of cohort and consortium/project names in sequencing-based GWAS publications (related to Figure 3). This table does not distinguish between cohorts (e.g. Old Order Amish Study) or consortia/projects (e.g. TOPMed) because this distinction is typically not made by authors. All instances were extracted, for example 'the JHS cohort sequencing by the TOPMed program', is represented as one instance of JHS, and one instance of TOPMed.

Supplemental References

1. Morales, J. *et al.* A standardized framework for representation of ancestry data in genomics studies, with application to the NHGRI-EBI GWAS Catalog. *Genome Biol.* **19**, 21 (2018).
2. Hulsen, T., de Vlieg, J. & Alkema, W. BioVenn - a web application for the comparison and visualization of biological lists using area-proportional Venn diagrams. *BMC Genomics* **9**, 488 (2008).
3. Lee, K. *et al.* Scaling up data curation using deep learning: An application to literature triage in genomic variation resources. *PLoS Comput. Biol.* **14**, e1006390 (2018).
